# Ribavirin for treating Lassa fever: A systematic review of pre-clinical studies and implications for human dosing

**DOI:** 10.1371/journal.pntd.0010289

**Published:** 2022-03-30

**Authors:** Alex P. Salam, Alexandre Duvignaud, Marie Jaspard, Denis Malvy, Miles Carroll, Joel Tarning, Piero L. Olliaro, Peter W. Horby

**Affiliations:** 1 Centre for Tropical Medicine and Global Health, Nuffield Department of Medicine, University of Oxford, Oxford, United Kingdom; 2 United Kingdom Public Health Rapid Support Team, London, United Kingdom; 3 Department of Infectious Diseases and Tropical Medicine, Division of Tropical Medicine and Clinical International Health, CHU de Bordeaux, Bordeaux, France; 4 UMR1219, INSERM, French National Research Institute for Sustainable Development (IRD), and University of Bordeaux, Bordeaux, France; 5 Programme PAC-CI/ANRS Research Center, CHU de Treichville, Abidjan, Côte d’Ivoire; 6 Alliance for International Medical Action, Dakar, Senegal; 7 Wellcome Center for Human Genetics, University of Oxford, Oxford, United Kingdom; 8 Mahidol Oxford Tropical Medicine Research Unit, Faculty of Tropical Medicine, Mahidol University, Bangkok, Thailand; National Institute of Allergy and Infectious Diseases Division of Intramural Research, UNITED STATES

## Abstract

Ribavirin is currently the standard of care for treating Lassa fever. However, the human clinical trial data supporting its use suffer from several serious flaws that render the results and conclusions unreliable. We performed a systematic review of available pre-clinical data and human pharmacokinetic data on ribavirin in Lassa. In in-vitro studies, the EC50 of ribavirin ranged from 0.6 μg/ml to 21.72 μg/ml and the EC90 ranged from 1.5 μg/ml to 29 μg/ml. The mean EC50 was 7 μg/ml and the mean EC90 was 15 μg/ml. Human PK data in patients with Lassa fever was sparse and did not allow for estimation of concentration profiles or pharmacokinetic parameters. Pharmacokinetic modelling based on healthy human data suggests that the concentration profiles of current ribavirin regimes only exceed the mean EC50 for less than 20% of the time and the mean EC90 for less than 10% of the time, raising the possibility that the current ribavirin regimens in clinical use are unlikely to reliably achieve serum concentrations required to inhibit Lassa virus replication. The results of this review highlight serious issues with the evidence, which, by today standards, would be unlikely to support the transition of ribavirin from pre-clinical studies to human clinical trials. Additional pre-clinical studies are needed before embarking on expensive and challenging clinical trials of ribavirin in Lassa fever.

## Author summary

Lassa fever is a viral disease endemic to West Africa that has been highlighted by the World Health Organisation as one of the top ten epidemic threats to global health. Ribavirin has been used to treat Lassa fever for more than 30 years. However, the clinical evidence for ribavirin is poor. We conducted a systematic review of the pre-clinical and human pharmacokinetic data in Lassa. We found that the minimum concentrations of ribavirin that inhibited Lassa virus replication varied hugely according to the experimental design and the type of cell and Lassa virus strain used. There was no comprehensive data on the concentrations of ribavirin in the blood of Lassa fever patients. Modelling of ribavirin concentration data from healthy humans suggest that the doses of ribavirin currently used to treat Lassa fever patients do not reliably achieve the concentrations required to inhibit viral replication.

## Introduction

Lassa fever is an acute viral disease caused by Lassa virus (LASV) that can lead to a viral haemorrhagic fever syndrome in some patients. It is endemic in several West African countries, including Nigeria, Sierra Leone, Guinea and Liberia [1]. As a result of its mortality, paucity of therapeutic and preventative interventions, and epidemic potential, Lassa fever has been highlighted by the World Health Organisation (WHO) as one of the top ten global epidemic threats [2,3]. Intravenous ribavirin is the therapeutic standard of care for Lassa fever. Ribavirin is part of national treatment guidelines in endemic countries [4], is recommended by the WHO, is on the WHO list of essential medicines for viral hemorrhagic fevers, and has been in use for over 30 years [5]. It is the only drug therapy currently used to treat Lassa fever.

The evidence base for ribavirin in Lassa fever primarily relies on one clinical trial conducted in the early 1980s in Sierra Leone [6]. This trial concluded that ribavirin reduced mortality in Lassa fever and should be used in all patients [6]. However, this trial suffered from several serious flaws that render the results and conclusions of the trial unreliable [7]. More recent analysis of previously unavailable data from a larger cohort of Lassa fever patients in Sierra Leone suggests that ribavirin may actually increase mortality in some patients [7]. Two recent systematic reviews both came to the same conclusion, that there is insufficient evidence to support the routine use of ribavirin in Lassa fever patients and that there is an urgent need to re-evaluate the use of ribavirin in Lassa fever [8,9]. However, before embarking on expensive and logistically complex trials of ribavirin in Lassa fever, it is worth reviewing the pre-clinical data supporting the use of ribavirin in Lassa fever in order to help design and inform such trials. Here, we present and discuss the results of a systematic review of the pre-clinical data for ribavirin in Lassa fever as well as human pharmacokinetic (PK) data. The results of our review highlight that the data suffer from several serious limitations, which need to be addressed prior to any clinical trials of ribavirin or before trials of new anti-viral therapies in which ribavirin is used as control therapy.

## Methods

### Search terms

Pubmed and Embase were searched using the search term: Lassa AND (ribavirin OR virazole), up until the 27th of July 2021. There were no language restrictions. Non-English language articles were translated using a professional translation service. Two authors independently screened article titles and abstracts, reviewed full text articles, and extracted data. A third author resolved any disagreements. Articles were assessed according to specific domains. Here we define “pre-clinical” data as in-vitro data and animal efficacy and PK data. We also investigated human PK studies as the PK and pharmacodynamics (PD) of ribavirin in Lassa fever patients are directly relevant to questions of dosing and target concentrations. The following inclusion criteria were applied for each domain:

- In-vitro data: studies presenting original data on the effect of ribavirin on LASV replication in-vitro. We excluded studies that used minigenome-based assays, though we did include studies that used recombinant LASV expressing fluorescent protein.
- Animal efficacy data: studies presenting original data on the effect of ribavirin on survival in animals experimentally infected with LASV in comparison to placebo or no treatment. We restricted articles to only those that investigated ribavirin monotherapy.
- Mechanism of action data: studies presenting original in-vitro or in-vivo data on the mechanism of action by which ribavirin acts in Lassa fever.
- Animal PK data: studies presenting original data on ribavirin concentrations in animals experimentally infected with LASV.
- Human PK data: studies presenting original data on ribavirin concentrations in humans with laboratory confirmed Lassa fever.

### Data extraction

For each article, the following data were extracted: first author, year of publication, and PMID number. In addition, the following data were extracted for each specific domain:

- In-vitro studies: LASV strain, cell type, multiplicity of infection (MOI), timing and duration of ribavirin exposure, timepoint at which viral replication was assessed, EC50 (concentration of ribavirin resulting in a 50% reduction in viral replication), EC90 (concentration of ribavirin resulting in a 90% reduction in viral replication), CC50 (concentration of ribavirin resulting in a 50% reduction in cell viability), SI50 (selectivity index, a value that indicates the relationship between the compound’s effective and toxic concentrations, calculated as CC50/EC50). Where EC50/90 were expressed as μM, they were converted into μg/ml.
- Animal studies: LASV strain, animal model, inoculation dose and route, sample size in ribavirin group, sample size in control group, dose and route and duration of ribavirin, time from inoculation to starting ribavirin (days), prophylaxis versus treatment (for the purpose of this review we considered treatment as initiation of ribavirin after symptom onset and prophylaxis as initiation of ribavirin before symptom onset since initiating drug therapy after symptom onset is more clinically relevant in humans as “treatment”), number of deaths in each group, whether ribavirin extended time to death, reduced peak viral titers, and reduced aspartate aminotransferase (AST), and any toxicity issues. We only extracted data on groups that received ribavirin monotherapy. Data were not extracted on groups that received ribavirin in combination with another antiviral.
- Mechanism of action data: LASV strain, human or animal data, study type (if human), sample size, experimental method, summary of findings.
- Animal PK data: LASV strain, animal model, experimental groups (i.e. infected and non-infected) dose, frequency, route and duration of ribavirin, matrix for concentration measurement, PK findings.
- Human PK data: country, experimental groups (i.e. infected and healthy), study type (e.g. cohort, series), LASV strain, sample size, dose, frequency, route and duration of ribavirin, matrix for concentration measurement, PK findings.

### PK modelling

PK modelling was carried out to determine the concentration profiles of the different ribavirin regimens currently in use to treat Lassa fever patients in order to compare against the in-vitro EC50/90 data. PK parameters were based on those provided by Preston et al [10]. A three-compartment disposition model, with bolus injection, was used for concentration-time profile simulations. The three different regimens were the McCormick regimen (loading dose of 33 mg/kg followed by 16 mg/kg QDS from day 1 to day 4, and then 8 mg/kg from day 5 to 10 [6]), the Irrua non-pregnant regimen (loading dose of 100 mg/kg followed by 25 mg/kg OD from day 2 to 7 and then 12.5 mg/kg day 8 to 10 in non-pregnant adults), and the Irrua pregnant regimen (loading dose of 100 mg/kg followed by 16 mg/kg QDS from day 2 to 5 and then 8 mg/kg TDS from day 6 to 10 in pregnant adults [4]).

## Results

Pubmed returned 157 results and Embase returned 336 results; 350 articles remained after removal of duplicates. Eleven studies presented in-vitro data (Table 1), eight animal data (Table 2), two mechanism of action data, and one human PK data. No study reported animal PK data.

**Table 1:** Summary of in-vitro data.

AUTHOR	YEAR	STRAIN	CELL	MOI	Ribavirin exposure	TIME ASSESSED	EC50	EC90 μg/mL	CC50	SI
Jahrling [20]	1980	Josiah	Vero	NA	With inoculation	96 hours	NA*	NA	NP	NP
			RAM	NA	With inoculation	96 hours	NA*	NA	NA	NP
Oestereich [81]	2016	Ba366	Vero	0.01	1 hr post inoculation	72 hours	6.47 μg/ml	8.22	NA^#^	
**Mudhasani [82]**	2015	Josiah	Hela	0.1	2 or 16 hr pre inoculation	24 hours	2.31 μg/ml	NA	> 49	> 21
Günther [15]	2004	AV	Vero	0.01	1 hr post inoculation	48 hours	9 μg/ml	14 μg/ml	> 35 ug/ml	NA
Caì [14]	2018	Josiah	Vero	0.1	NA	48 hours	NA	7.27 μg/ml	NA	NA
		eGFR Josiah	A549	0.1	NA	48 hours	4.05 μg/ml		NA	NA
				0.1	“	72 hours	9.86 μg/ml		“	“
			Hela	0.1	“	48 hours	1.20 μg/ml		“	“
				0.1	“	72 hours	2.29 μg/ml		“	“
			Huh7	0.1	“	48 hours	3.37 μg/ml		“	“
				0.1	“	72 hours	8.45 μg/ml		“	“
			Vero	0.1	“	48 hours	8.65 μg/ml	1.50 μg/ml^	“	“
				0.1	“	72 hours	21.72 μg/ml		“	“
Tong [83]	2018	Josiah	Vero	0.1	1 hr post inoculation	NA	NA**	NA	NA^#^	NA
**Kim [84]**	2019	eGFR Josiah	A549	0.1	2 hr pre inoculation	48 hours	NA	NA	NA	NA
**Mudhasani [85]**	2014	Josiah	Hela	1	2 hr pre inoculation	24 hours	1.66 ug/ml	NA	NA	> 29.4
Olschlager [16]	2011	AV	Vero	0.01	1 hr post inoculation	48 hours	16 ug/ml	29 ug/ml	NA^#^	NA
Petkevich [86]	1981	Josiah	Vero	NA	NA	72–96 hours	NA***	NA	NA^#^	NA
Welch [13]	2016	eGFR Josiah	Huh7	0.1	1 hr post inoculation	48 hours	0.60 ug/ml	NA	> 12 ug/ml	> 4.88

Ribavrin exposure refers to the addition of ribavirin relative to infection of cells with Lassa virus. Time assessed refers to the time point at which viral titres were assessed afterinfection. Hr = hour. RAM = Rhesus alveolar macrophages. NA = not available. NP = not performed.

* 50 μg/ml ribavirin completely inhibited replication from -3.3 log10 in vero cells and from -1.3 log10 in Rhesus alveolar macrophages.

** 33uM reduced virus titer by 0.5 log10 pfu/mL and 100uM reduced virus titer by 1.7 log10 pfu/mL relative to controls.

*** 50ug/ml reduced viral titre from 3.3 to 1.7 x 10(3) PFU/ml. 100 ug/ml and 200 ug/ml completely inhibited.

^#^did not significantly affected cell viability. ^ unclear why the EC90 is lower than the EC50.

**Table 2:** Percentage of time above the lower and upper range of the EC50/90 and above the EC50/90 means for the different ribavirin regiments based on PK modelling.

Time above target (%)	EC50			EC90		
	0.6	7	22	7	15	29
**24hr** **McCormick** Irrua non-pregnant Irrua pregnant	100 100 100	12.5 7.0 10.5	1.9 3.8 4.6	12.5 7.0 10.5	5.5 4.7 6.3	1.0 3.2 3.5
**48hr** **McCormick** Irrua non-pregnant Irrua pregnant	100 100 100	13.2 5.7 12.5	1.7 2.5 3.2	13.2 5.7 12.5	5.3 3.4 5.8	0.5 1.9 1.8
**72hr** **McCormick** Irrua non-pregnant Irrua pregnant	100 100 100	14.3 5.2 14.1	1.8 2.1 2.8	14.3 5.2 14.1	5.5 2.9 5.9	0.3 1.5 1.2

**Fig 1:**
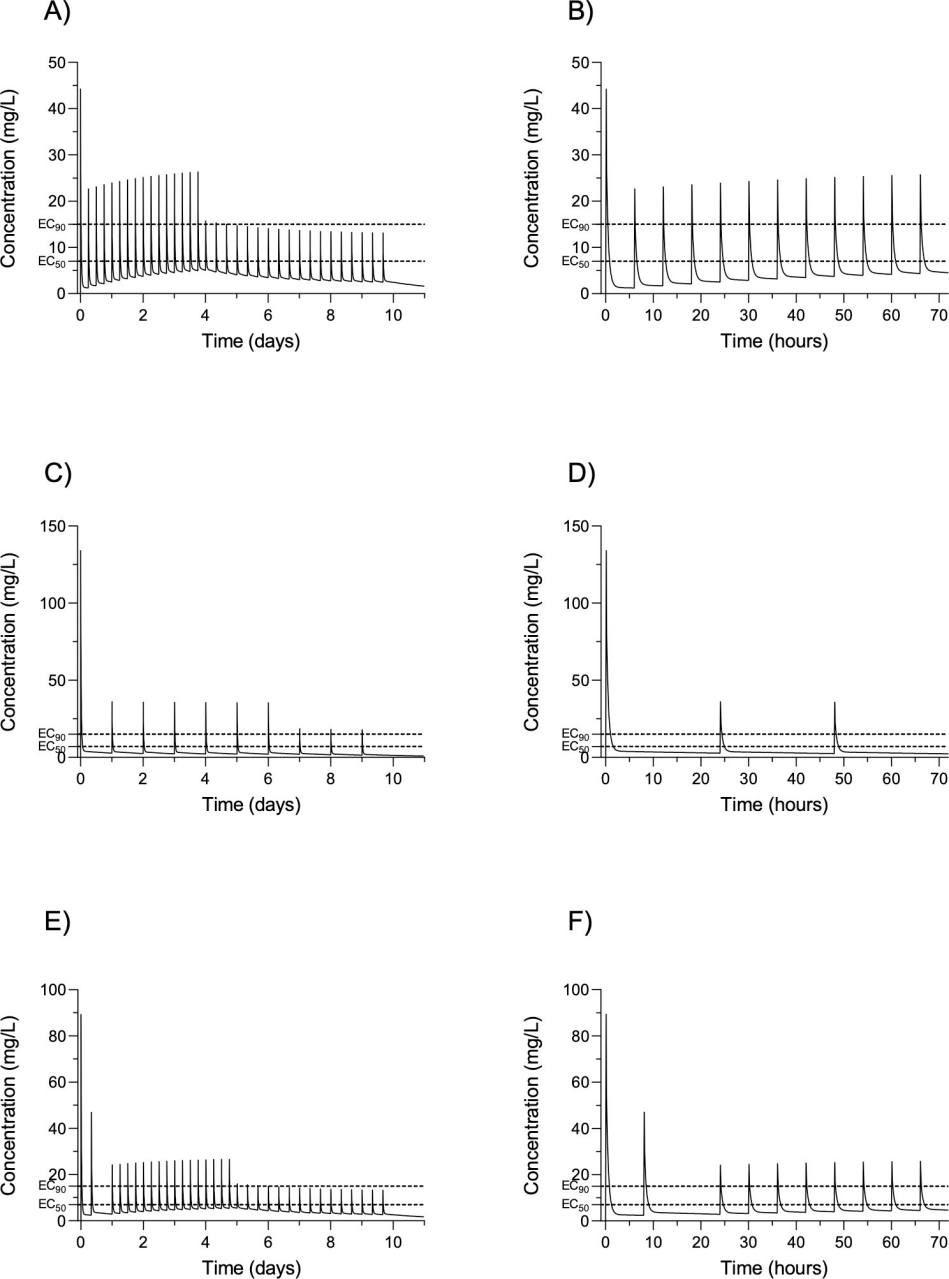
Pharmacokinetic simulations of the different ribavirin regimens. A) McCormick regimen day 0–10 and B) 0–72 hours. C) Irrua non-pregnant regimen day 0–10 and D) 0–72 hours. E) Irrua pregnant regimen day 0–10 and F) 0–72 hours. Dash lines represent mean EC50 and mean EC90.

All eleven in-vitro studies demonstrated an inhibitory effect of ribavirin on LASV replication, although the concentration required to demonstrate an effect varied considerably across experiments and as a result of MOI, LASV strain, cell type, and time point after infection at which viral replication was assessed (Table 1), as well as the timing and duration of ribavirin exposure. LASV strains used were Josiah (6), eGFR Josiah (2), AV (2), and Ba366 (1). Cell types were Vero (10) (an immortalised African green monkey kidney epithelial cell line), Rhesus alveolar macrophage (1), Hela (1) (an immortalised human cervical cancer cell line), A549 (1) (an immortalised human alveolar adenocarcinoma basal epithelial cell line), and Huh7 (2) (an immortalised human hepatocyte carcinoma cell line). There were no in-vitro data on the effect of ribavirin against LASV in human macrophages, human dendritic cells, human monocytes, or human endothelial cells, the cell types thought to be the most commonly infected by LASV in humans [11,12]. The EC50 of ribavirin ranged from 0.6 μg/ml to 21.72 μg/ml and the EC90 from 1.5 μg/ml to 29 μg/ml. The EC50 was consistently higher in Vero (primate) cells relative to other (human) cells. Two studies used the same experimental setups of Josiah eGFR LASV, Huh7 cells, MOI of 0.1, and timepoint at which viral replication was assessed of 48 hours–one reported an EC50 of 0.60 μg/ml (Welch et al)[13], the other an EC50 of 3.37 μg/ml (Caì et al)[14], though it is unclear if these two studies used the same timing of ribavirin exposure. Two studies used the same experimental setups of AV LASV, Vero cells, MOI of 0.01, timing of ribavirin exposure, and same timepoint at which viral replication was assessed of 48 hours–one reported an EC50 of 9 μg/ml and EC90 of 14 μg/ml (Günther et al) [15], the other an EC50 of 16 μg/ml and EC90 of 29 μg/ml (Olschlager et al) [16]. One paper reported a lower EC90 (1.50 μg/ml) than EC50 (8.65 μg/ml) with eGFR Josiah at 48 hours and an MOI of 0.1 (Caì et al)[14]. This is likely to represent a typographical error in the paper, since an EC50 of 1.5 results in a predicted EC90 of 8.6 (using an approximate hill coefficient of 0.8)[17]). The EC50 range was 0.6–21.72 μg/ml, with a mean of 6.8 μg/ml and the EC90 range (discounting the 1.50 from Caì et al) was 7.27–29 μg/ml, with a mean of 14.6 μg/ml. Fig 1A–1F are the PK profiles of the three main ribavirin regimens in use in patients, whilst Table 2 presents the time above the ranges and means of EC50/90 for the different regimens within the first 72 hours of starting therapy. Except for the lower range of the EC50 (0.6 μg/ml), each of the three regimens result in little time spent above the EC50/90.

All eight studies that presented animal data investigated the effect of ribavirin as post-exposure prophylaxis, but only two [18,19] amongst these investigated the effects of ribavirin as treatment (where the initiation of ribavirin was clearly defined in the article as having started after the onset of symptoms) (Table 3). Most studies used Josiah strain (7/8). Only one study used the aerosolised route of inoculation, whilst others either used intramuscular, subcutaneous or intraperitoneal routes and there were variations in inoculation dose. Samples sizes were small. Doses of ribavirin ranged from 20mg/kg to 230mg/kg for loading doses and 20mg/kg to 90mg/kg total daily maintenance doses in non-human primates (NHP), and from 25mg/kg to 160mg/kg total daily dose in rodents. There was no indication in any of these studies as to how these doses of ribavirin were derived, and there were no studies (within the domain of “Animal outcome” or “Animal PK”) on ribavirin PK in animal models of Lassa fever. In the NHP, prophylactic use of ribavirin resulted in 100% survival [18,20], 75% survival [21], 60% [19], and 0% survival [22] across the five studies (all Josiah strain). In the treatment NHP studies, ribavirin resulted in an approximate survival rate of 50% in one study (20mg/kg loading followed by 10mg/kg BD) [19], and survival rates of 50% at 75mg/kg loading followed by 15mg/kg and 0% at 230mg/kg loading followed by 45mg/kg BD in the other study [18]. Death at higher doses appeared to be due to severe anaemia [18]. In rodents, pre-exposure prophylaxis with ribavirin monotherapy had no effect on survival. There were no rodent studies that examined the effects of ribavirin treatment (as far as it was possible to deduce from the data and text as clearly having been started after symptom onset).

**Table 3:** Summary of animal data.

PMID	YEAR	STRAIN	ANIMAL	RIBAVIRIN DOSE	RIBAVIRIN STARTED	DEATHS RIBAVIRIN	DEATHS CONTROLS	VIRAL TITRES	AST	TIME TO DEATH	TOXICITY
**PROPHYLAXIS**											
Jahrling [20]	1980	Josiah (1.2x10^6^ pfu SC)	Rhesus macaques	50 mg/kg loading, then 10 mg/kg tds 50 mg/kg,loading, then 10 mg/kg tds	Day 0 Day 5	0 (4) 0 (4)	6 (10)	↓ ↓	↓ ↓	NC NC	NR NR
Jahrling [18]	1984	Josiah (1.2x10^6^ pfu SC)	Cynomolgus macaques	75mg/kg loading, then 15mg/kg bd 75mg/kg loading, then 15mg/kg bd	Day 0 Day 4	0 (4) 0 (4)	13 (14)	↓ ↓	NA NA	NC NC	Anaemia Anaemia
Dvoretskaia [19]	1990	Josiah (15–20 pfu AR)	Baboon hamadryas	Loading 20mg/kg, then 10mg/kg bd	Day 0	2 (5)	4 (4)	↓	NA	↑	NR
Fidarov [21]	1990	Josiah (3000 pfu IM)	Japanese macaques	Loading 75mg/kg, then 15mg/kg bd	Day 0	1 (4)	4 (4)	↓	NA	↑	NR
Cashman [87]	2011	Josiah (1000 pfu SC)	Hartley guinea pigs	25 mg/kg od	Day 0	8 (8)	7 (7)	↓	-	↑	NR
Safronetz [60]	2015	Josiah^ (10^5^ TCID50 IP)	Hartley guinea pigs	50mg/kg od	Day 2	9 (9)*	6 (6)	↓	NA	↑	NR
Oestereich [81]	2016	Ba366 (1000 pfu IP)	*Ifnar*–/–B6 mice	80mg/kg od 80mg/kg od 80mg/kg bb	Day 0 Day 4 Day 4	5 (5) 5 (5) 4 (5)	8 (8)	- - -	↓ ↓ ↓	↑ - ↑	NR NR NR
**Lingas [22]**	2021	Josiah (10^4^ TCID50 IM)	Cynomolgus macaques	Loading 30 mg/kg, then 10 mg/kg tds Loading 30 mg/kg, then 30 mg/kg od	Day 4 Day 4	4 (4)** 4 (4)**	8 (8)**	↓ ↓	NA NA	↑ ↑	NR NR
**TREATMENT**											
Jahrling [18]	1984	Josiah (1.2x10^6^ pfu SC)	Cynomolgus monkeys	75mg/kg loading, then 15mg/kg BD 150mg/kg loading, then 30mg/kg BD 230mg/kg loading, then 45mg/kg BD	Day 7 Day 7 Day 7	4 (8) 3 (4) 6 (6)	13 (14)	↓ NA NA	NA NA NA	↑ ↑ ↑	Anaemia Anaemia Anaemia
Dvoretskaia [19]	1990	Josiah (15–20 pfu AR)	Baboon hamadryas	Loading 20mg/kg, then 10mg/kg BD	When feverish	4 (9)	4 (4)	↓	NA	↑	NR

NA not available. NC not applicable. NR none reported. SC subcutaneous. IM intramuscular. IP intraperitoneal. AR aerosolised. ↓ Reduced. ↑ Increased.–Unchanged.

* 3 of the 9 were euthanised due to clinical severity.

** Some animals euthanised due to clinical severity, numbers not indicated.

^ Guinea pig adapted Josiah strain.

Only one study was identified which included human PK data in Lassa fever [23]. In this study, 5 patients received intravenous ribavirin and 4 received oral ribavirin. A competitive binding radioimmunoassay was used to measure ribavirin concentrations. Patients received either 1000mg QDS IV for 3 days or 1000mg/day PO for 10 days. Samples were collected 2.5 hours post dose, although it was unclear after which dose samples were collected, on which days, and how many samples were collected in total. Ribavirin concentrations ranged from 0.3–2.3 μg/ml (mean 0.8 μg) in the oral group and from 1.1–16.9 μg /ml in the IV group (mean 7.8 μg/ml). No PK parameters were presented due to the sparsity of the concentration data.

Two studies were identified which investigated the mechanism of action of ribavirin in Lassa. One in-vitro study investigated the effect of GTP depletion (a recognized mechanism of action of ribavirin against some other viruses via inhibition of inosine monophosphate dehydrogenase) on LASV replication [16]. Whilst ribavirin did inhibit viral replication, restoring the intracellular GTP pool by exogenous addition of guanosine had no effect on ribavirin’s antiviral activity. The other study used mathematical models and experimental data in LASV-infected mice treated with ribavirin alone or in combination with favipiravir to explore different mechanisms of action for ribavirin [24]. Ribavirin appeared to protect infected cells from dying, thereby reducing the release of cell damage markers in the circulation, rather than impairing viral transmission, viral production, or enhancing the host’s immune response.

## Discussion

This systematic review reveals that the pre-clinical evidence-base for ribavirin use in the treatment of Lassa fever is conflicting and is largely insufficient by modern standards to warrant human trials in Lassa fever. Furthermore, no dose justification for the current ribavirin treatment regimens can be found.

The in-vitro data reviewed here show a wide range of EC50/90 for ribavirin against LASV, within and between different studies, even with similar experimental setups, and variations in EC50/90 according to cell type, MOI, and timing of ribavirin exposure. There was a 35-fold difference between the lowest and the highest EC50 (range 0.6–21.72 μg/ml) and a 19-fold difference between the lowest and the highest EC90 (range 1.5–29 μg/ml) (this lower range of the EC90 should be interpreted with caution given that the EC90 was lower than the EC50 in the study which reported this). Based on PK modelling, as can be seen from Table 2 and Fig 1A–1F, these regimens do not come close to superseding the mean and upper range of EC50/90 for any significant amount of time.

Whilst all in-vitro studies demonstrated that ribavirin inhibited LASV replication, none of these studies used the human cell types thought to be most commonly infected by LASV and important in driving the pathophysiology of Lassa fever, namely macrophages, dendritic cells, monocytes and endothelial cells [11,12]. Whilst ribavirin has been shown to have broad spectrum antiviral effects across different viruses [25,26], these affects vary (and can be present or absent) according to the cell type, virus, concentration of ribavirin, and timing and duration of ribavirin exposure. Specifically, some cell types appear to be naturally resistant to the antiviral effects of ribavirin. In some cells this appears to be due to reduced intracellular uptake of ribavirin as a result of decreased or absent transporter activity [27], whilst in others it appears to be due to differences in the intracellular metabolism of ribavirin [28]. For example, Shah et al showed that ribavirin inhibited vesicular stomatis virus (VSV) and Sendai virus (SV) in HeLa, HEp2 (human epidermal carcinoma cells) and BSRT7 cells (cells which are derived from BHK21) but had little effect on VSV and SV in BHK21 (Syrian golden hamster kidney fibroblast cells), Vero and A549 cells [28]. This effect was consistent across different ribavirin concentrations and exposures, and different MOI. This resistance to ribavirin’s antiviral activity was not as a result of decreased uptake across the different cell types–all cell types showed similar levels of ribavirin uptake. Nor did this effect appear to be due to any differences in viral growth phenotype in different cells. Neither was there any suggestion of the emergence of viral resistance to ribavirin. However, the different cell types did show dramatic differences in the long-term accumulation of ribavirin. Specifically, BHK21, Vero and A549 cells showed markedly decreased levels of ribavirin accumulation, indicating differences in intracellular metabolism. In our review, we identified the EC50 of ribavirin for LASV as being consistently higher in Vero cells relative to other cells. More generally, ribavirin has effects on cellular physiology independent of viral infection, and this effect depends again on ribavirin exposure and cell type. For example, ribavirin can either have no effect, increase, or decrease macrophage nitric oxide production depending on the concentration, timing and duration of exposure [29,30]. Nitric oxide is thought to play a key role in the vascular pathophysiology of LASV [31,32]. Therefore, overall, it should not be assumed that ribavirin has anti-LASV effects in human mononuclear cells and/or endothelial cells in general or at equivalent doses to those currently used in humans without direct evidence, which is currently lacking. This needs to be tested experimentally. Going forward, standardization of experimental protocols more generally for in-vitro studies of anti-LASV agents would also be extremely helpful for comparison within and between agents.

The single published PK study of ribavirin in Lassa fever does not present sufficient ribavirin concentration data to be able to estimate ribavirin PK parameters in the context of Lassa fever [23]. The wide range of different EC50/90 also make it unclear which concentrations of ribavirin to aim for in any phase 2 PK/PD ribavirin modelling studies, a necessary step which has been suggested prior to any future trials of ribavirin [33]. However, as can be seen from Fig 1A–1C, which is a PK model of serum ribavirin concentrations using the “McCormick dosing” regimen based on PK parameters obtained from phase I studies of ribavirin in healthy volunteers [10], the plasma concentration of ribavirin falls rapidly after administration. According to this model, concentrations are not maintained above the majority of the documented EC50, let alone EC90, for most of the time during therapy. The findings are similar for modelling of the Irrua regimens. Concentrations probably need to be maintained above the EC90, and for most of the time (e.g. > 80% of the time), at least early on in treatment, for effective, rapid, and sustained viral suppression. However, ribavirin is a pro-drug and its antiviral effects are mediated by one or more of its active metabolites (ribavirin monophosphate, diphosphate and triphosphate) [34]. Therefore, likely of more relevance are the intracellular concentrations of ribavirin’s metabolites, specifically in human mononuclear cells and/or endothelial cells. Indeed, this may be another explanation for why there is variation in the EC50/90 of ribavirin in the different experimental setups. The relationship between serum ribavirin concentrations and cellular metabolite concentrations depends, in part at least, on cell type and the differential expression of the enzymes responsible for the uptake and conversion of ribavirin into its metabolites as well the metabolism of the metabolites themselves. For example, Page et al found the ratio of ribavirin monophosphate, diphosphate and triphosphate to be 4:1:40 in human fibroblasts, 3:1:8 in lymphoblasts and 1:5:17 in erythrocytes [35]. Furthermore, different mechanisms of action have been proposed and investigated for the different ribavirin metabolites across different viruses [36]. Therefore, robust in-vitro data are needed on the EC50/90 of ribavirin as well as its metabolites in LASV-infected human mononuclear cells and/or endothelial cells. Future human PK/PD studies should also therefore involve the measurement of intracellular ribavirin metabolite concentrations in peripheral blood mononuclear cells (PMBC).

In both the in-vitro and animal studies, the most frequently used strain of LASV was Josiah. Josiah was isolated in Sierra Leone [37], whilst AV was identified in Côte d’Ivoire [38] and Ba366 in Guinea [39]. LASV has multiple different strains across seven distinct lineages. Lineages I-III and VI exist in Nigeria, whilst IV, V and VII exist in other West African countries [37–41]. Nigeria experiences by far the highest annual number of cases of Lassa fever in comparison to other West African countries, and yet there exists no in-vitro data on the effect of ribavirin on LASV replication from Nigerian lineages (nor animal data). There is high nucleotide sequence diversity across the different LASV lineages and strains [42]. It has been hypothesised that LASV strain may impact disease phenotype and severity in humans [43], and some animal data using different strains at identical doses and routes of inoculation do show differences in disease phenotype and outcome [44,45]. Although there were large differences in EC50/90 according to LASV strain in the different in-vitro studies we reviewed, there were no two studies that used the same combination of cell type, MOI, and timing of ribavirin exposure, but different LASV strains. Therefore, it is unknown whether the differences in EC50/90 between studies with different strains is potentially a result of LASV strain or the other described factors. This is worth investigating experimentally. It is not inconceivable that ribavirin may impact different LASV strains differently given the various hypothesised antiviral mechanisms of action of ribavirin (see below). There is up to 25% sequence variation in the LASV small segment (S) and 32% sequence variation in the large (L) segment across LASV lineage genomes [42,46]. The L segment encodes the viral RNA-dependent RNA polymerase [47], inhibition of which is one proposed mechanism of action of ribavirin [48,49]. The S segment encodes the nucleoprotein (NP). NP participates in the suppression of host interferon-type-I, as does the Z protein (encoded by L), both of which allow LASV to evade host immune sensing [50–52]. Data exists showing differences in the ability of NP across different strains to differentially inhibit hRIG-I function (an innate antiviral immune protein involved in interferon-type-I production) [53]. Modulation of interferon-stimulated gene responses is another hypothesised mechanism of action of ribavirin in viral infections [54,55]. In summary, data showing differences in ribavirin EC50/90 (and indeed other pre-clinical studies of compounds with antiviral activity against LASV) across different LASV strains might have important implications for Lassa fever treatment and implications for the choice of ribavirin dosing to test in an international clinical trial of ribavirin across different countries in West Africa. Such an international trial is preferred to a trial in a single country because of the sample sizes required. If LASV strain does impact ribavirin EC50/90, this could introduce significant heterogeneity.

The animal data supporting ribavirin as a treatment against symptomatic LASV disease have significant limitations. Only two studies clearly identified ribavirin being started after symptom onset, whilst the other six studies investigated ribavirin as prophylaxis (of which three were rodent models of Lassa—it is unclear how well these rodent models recapitulate human disease). There are many examples of anti-infective agents having a prophylactic effect on survival in animal models (or in-vitro for that matter and then no effect in animal models) but little or no effect on survival when started after symptom onset, for example favipiravir in the context of Ebola [56], and hydroxychloroquine and lopinavir/ritonavir in the context of COVID-19 [57,58]. Notably, in Lassa fever patients the median duration from symptom onset to presentation at a health care facility is approximately 6 days (IQR 4–10 days) and the median duration to drug administration is approximately 8 days (IQR 7–13 days) [59]. The different NHP studies used different species of NHP, routes of inoculation of LASV, and different doses of ribavirin. Therefore, direct comparison between studies is not possible. The four NHP prophylaxis studies performed in 1980/90s showed a survival benefit whereas the one recent study [22] showed no survival benefit at all. This recent study used a similar dosing regimen as McCormick et al (30 mg/kg loading, then 10mg/kg TDS [6]), despite the fact the equivalent conversion dose in NHP would be substantially higher. This is a markedly lower loading dose than the 1980/90s studies (apart from the one study which used the aerosolized route of inoculation). The NHP model used in the recent study and the NHP models used in the 1980/90s appear to have similar disease course, which approximates that seen in humans (namely hepatitis, renal failure, respiratory distress and shock). Therefore, it is possible, though unclear (there were also differences in LASV inoculation dose and route), that the difference in survival is due to the lower dosing regimen. In contrast to several of the NHP studies, the prophylactic studies in rodents showed no effect of ribavirin on survival. Although in one of these rodent studies, guinea pigs treated with ribavirin were free of symptoms whilst ribavirin was continued but only succumbed to infection when ribavirin treatment ended. It is unclear whether continuing ribavirin in this specific study would have resulted in survival [60]. These rodent studies either used genetically modified rodents or required adaptation of the LASV to recapitulate severe disease, and it is unknown what effect this has on the drug efficacy for the reasons described above. Further, given that some cell types appear resistant to ribavirin’s antiviral effects, it cannot be assumed that the absence of an effect in rodents represents an equivalent absence of effect in other species. We did not identify any in-vitro studies in our review that investigated the antiviral activity of ribavirin against LASV in rodent cell lines.

Amongst the two therapeutic studies, one used the aerosolised route of inoculation with one dosing regime and the other used the intramuscular route with three different dosing regimens. A total of 27 NHP received ribavirin across these two studies. Survival benefits were not that impressive, with an approximately 50% survival rate in the lower dose groups and 0% survival in the higher dose groups. This is, again, in the context of ribavirin being started early on in the disease course (e.g. on the day of fever development). At the higher doses, ribavirin appeared to directly result in death due to severe haemolytic anaemia, rather than failure to prevent progression of disease due to Lassa fever. Haemolytic anaemia is a recognised side effect of ribavirin due to the absence of 5’-nucleotidase and alkaline phosphatase in erythrocytes and therefore the accumulation of RTP, which results in oxidative membrane damage [61,62]. Ribavirin treatment results in a severe drop in haemoglobin in approximately 20% of Lassa fever patients [63]. This, in the context of a disease where patients are prone to bleeding [63] and in healthcare settings where there is often limited availability of blood transfusion [64]. The effect of ribavirin on erythrocyte survival is dose cumulative—RTP accumulates in circulating erythrocytes until dysfunctional erythrocytes are removed from the circulation [61]. Many people suffer from anaemia in West Africa as a result of malaria and nutritional deficiencies [65], and the risk of ribavirin induced morbidity (and potentially mortality) secondary to anaemia is even higher in such a population. Importantly, approximately 30% of Lassa fever patients require blood transfusions as a result of anaemia and this is rarely secondary to frank haemorrhage [63]. Specifically, defining to what extent anaemia is due to ribavirin therapy would be important to accrue before any trial as this would have important safety considerations. Overall, the animal data supporting ribavirin in Lassa fever are very limited as well as conflicting. Whether such data would support taking ribavirin into clinical trials in humans by today’s regulatory standards, especially given toxicity concerns, is unclear.

Three different ribavirin regimens are used to treat Lassa fever in West Africa. The McCormick regimen, based on the one clinical trial that exists in Lassa fever (which notably suffers from numerous methodological flaws, including the use of historic controls, inclusion of pregnant women in the control group but not the ribavirin group, and post hoc merging of treatment groups [7]), uses a loading dose of 33 mg/kg followed by 16 mg/kg QDS from day 1 to day 4, and then 8 mg/kg TDS from day 5 to 10 [6]. Two other regimens are in use, primarily in Nigeria. The Irrua regimen uses a loading dose of 100 mg/kg followed by 25 mg/kg OD from day 2 to 7 and then 12.5 mg/kg day 8 to 10 in non-pregnant adults, and a loading dose of 100 mg/kg followed by 16 mg/kg QDS from day 2 to 5 and then 8 mg/kg TDS from day 6 to 10 in pregnant adults [4]. Some clinicians continue ribavirin beyond 10 days. There is no published data on how these three different ribavirin regimens were derived and it is unclear when the Irrua regimen began to be used in Nigeria. The total ribavirin dose across ten days is 417 mg/kg for the McCormick regimen, 287 mg/kg for non-pregnant Irrua regimen, and 476 mg/kg for the pregnant Irrua regimen. By comparison, the total dose across ten days in the Jahrling et al study [18] in Cynomolgus monkeys (in which ribavirin resulted in death) was 375 mg/kg at the lowest dose, 750 mg/kg at the medium dose and 1130 mg/kg at the highest dose, which, using FDA guidance [66], is equivalent to 122 mg/kg, 243 mg/kg, and 264 mg/kg respectively in humans. It is notable that the three different total ribavirin doses currently in use in humans exceed the NHP equivalent dose that resulted in severe haemolytic anaemia and 100% mortality. The highest dose used across the different regimens is in pregnant women (almost double the highest NHP equivalent). This regimen (which is without any basis) is in the context of there being no trial data for ribavirin in pregnant women at all (because they were excluded from the ribavirin arms but not the control arms in McCormick et al [6]) and in the context of ribavirin having known severe teratogenic effects [67,68]. This NHP data, and the recent analysis of previously unpublished human data suggesting that ribavirin may actually increase mortality in some Lassa patients [7] (see below), raise critical concerns about the safety of ribavirin in Lassa fever patients.

There are sparse data on any potential mechanisms of action of ribavirin in Lassa fever. Across multiple different viruses, numerous different antiviral mechanisms of action for ribavirin have been investigated and proposed [36], mediated by ribavirin’s metabolites (monophosphate, diphosphate and triphosphate). Broadly speaking these can be divided into inhibition of (A) viral replication via 1. inhibition of IMPDH [69], 2. inhibition of mRNA capping [70], 3. induction of host spermidine-spermine acetyltransferase [71], 4. binding to viral RNA-dependent RNA polymerases and chain termination [49], 5. promotion of viral mutagenesis and error catastrophe [72], or (B) modulation of cellular immune function via modulation of 1. TH1 and TH2 balance [73], 2. modulation of interferon stimulated gene expression (55). The only robust clinical data for ribavirin in any viral infection in humans is for hepatitis C, where ribavirin is only effective at inducing viral clearance in combination with interferon-α [74,75]. Its mechanism of action in humans with hepatitis C appears not to be antiviral [76], despite in-vitro data demonstrating robust inhibition of hepatitis C replication (49). Rather, its effect appears to be immune modulatory [77]. The in-vitro antiviral effect of ribavirin against other specific viruses has also failed to translate into clinical success in human trials [78–80]. In this review, we identified a rodent study which suggested that ribavirin limited hepatitis in Lassa fever via a cell protective effect [24]. It is noteworthy that analysis of the previously unpublished US Army Lassa fever cohort data raises the possibility that ribavirin may actually increase mortality in patients with hepatic aspartate aminotransferase < 150 UI/mL [7]. Only in patients with AST > 300 UI/mL did ribavirin appear to be protective (although this does need to be interpreted with caution because of possible bias). One possibility is that ribavirin’s effect in patients with high AST levels (if real) could be due to immune-modulatory or cell protective effects. Further data supporting a non-antiviral effect of ribavirin in Lassa fever would be important to accrue as this might be a rationale for the testing of combination therapy with an effective antiviral agent in clinical trials.

## Conclusion

This review summaries the available pre-clinical data on ribavirin in Lassa fever and highlights several critical issues, both in terms of efficacy and safety, which together with concerns regarding the clinical data in humans add extra credence to the proposition that the routine use of ribavirin in Lassa fever should be reconsidered. Convincing the medical community that the use of a drug that has been standard clinical practice for decades may in fact be ineffective, or indeed be harmful, is challenging. However, further data is needed to support the continued use of ribavirin in Lassa fever. Specifically, the following steps should ideally be taken before any expensive and challenging clinical trials in which ribavirin’s effectiveness is directly investigated or in which ribavirin is used as a control arm in trials of new antivirals: 1. in-vitro experiments investigating the antiviral activity of ribavirin and its active metabolites against LASV in human PBMC and/or endothelial cells across different LASV strains with standardization of MOI and timing of ribavirin exposure, 2. in-vitro experiments investigating the mechanism of action of ribavirin and its active metabolites in LASV infected human PBMC and/or endothelial cells, 3. at least one further in-vivo experiment in NHP investigating an equivalent ribavirin dosing regimen to that currently used in humans in West Africa 4. PK and PD studies of ribavirin in humans with Lassa fever investigating any correlations between ribavirin and ribavirin metabolite concentrations and viral load and expression of interferon stimulated genes and other relevant genes and proteins [88,89].

It is possible that we may be harming more patients than we are benefiting with the continued used of ribavirin in Lassa fever. Discussions need to be had about the continued use of ribavirin in Lassa fever and adequate investments made if the question of ribavirin’s effectiveness is to be answered definitively.
